# Visual analytics in cheminformatics: user-supervised descriptor selection for QSAR methods

**DOI:** 10.1186/s13321-015-0092-4

**Published:** 2015-08-19

**Authors:** María Jimena Martínez, Ignacio Ponzoni, Mónica F Díaz, Gustavo E Vazquez, Axel J Soto

**Affiliations:** Departamento de Ciencias e Ingeniería de la Computación, Laboratorio de Investigación y Desarrollo en Computación Científica (LIDeCC), Instituto de Ciencias e Ingeniería de la Computación (ICIC), Universidad Nacional del Sur, Av. Alem 1253, 8000 Bahía Blanca, Argentina; Planta Piloto de Ingeniería Química (PLAPIQUI)-UNS-CONICET, Co., La Carrindanga km.7, CC 717 Bahía Blanca, Argentina; Facultad de Ingeniería y Tecnologías, Universidad Católica del Uruguay, Av. 8 de Octubre 2801, CC 11300 Montevideo, Uruguay; Faculty of Computer Science, Dalhousie University, 6050 University Av., Halifax, Canada

**Keywords:** Feature selection, Visual analytics, QSAR, Cheminformatics

## Abstract

**Background:**

The design of QSAR/QSPR models is a challenging problem, where the selection of the most relevant descriptors constitutes a key step of the process. Several feature selection methods that address this step are concentrated on statistical associations among descriptors and target properties, whereas the chemical knowledge is left out of the analysis. For this reason, the interpretability and generality of the QSAR/QSPR models obtained by these feature selection methods are drastically affected. Therefore, an approach for integrating domain expert’s knowledge in the selection process is needed for increase the confidence in the final set of descriptors.

**Results:**

In this paper a software tool, which we named Visual and Interactive DEscriptor ANalysis (VIDEAN), that combines statistical methods with interactive visualizations for choosing a set of descriptors for predicting a target property is proposed. Domain expertise can be added to the feature selection process by means of an interactive visual exploration of data, and aided by statistical tools and metrics based on information theory. Coordinated visual representations are presented for capturing different relationships and interactions among descriptors, target properties and candidate subsets of descriptors. The competencies of the proposed software were assessed through different scenarios. These scenarios reveal how an expert can use this tool to choose one subset of descriptors from a group of candidate subsets or how to modify existing descriptor subsets and even incorporate new descriptors according to his or her own knowledge of the target property.

**Conclusions:**

The reported experiences showed the suitability of our software for selecting sets of descriptors with low cardinality, high interpretability, low redundancy and high statistical performance in a visual exploratory way. Therefore, it is possible to conclude that the resulting tool allows the integration of a chemist’s expertise in the descriptor selection process with a low cognitive effort in contrast with the alternative of using an ad-hoc manual analysis of the selected descriptors.

**Electronic supplementary material:**

The online version of this article (doi:10.1186/s13321-015-0092-4) contains supplementary material, which is available to authorized users.

**Graphical abstract:**
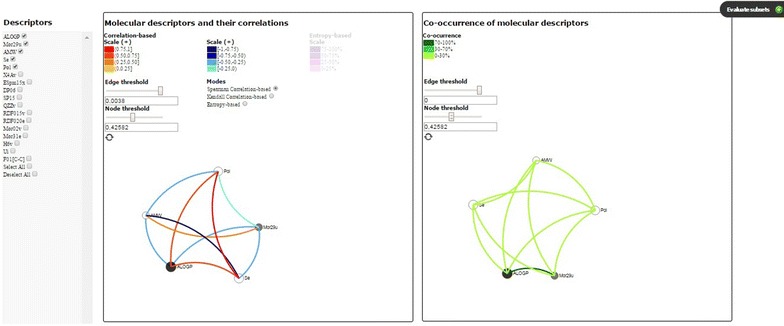
VIDEAN allows the visual analysis of candidate subsets of descriptors for QSAR/QSPR. In the two panels on the top, users can interactively explore numerical correlations as well as co-occurrences in the candidate subsets through two interactive graphs.

## Background

Quantitative structure–activity/structure–property relationship (QSAR/QSPR) models are regression or classification models widely used in cheminformatics, where a biological activity or a chemical property of chemical compounds are modeled in terms of their molecular descriptors. The design of QSAR/QSPR models requires dealing with several problems. One of them is the selection of the most relevant set of molecular descriptors for the property or activity that is intended to be modeled. Chemical structures are usually encoded by a variety of descriptor families such as functional groups, topological, constitutional, thermodynamic, quantum mechanical, etc. Several of them may contribute similar information or may be irrelevant for the biological activity under study, and thus, affecting the discovery of the descriptor-activity relationship. For this reason, the selection of the most relevant descriptors is regarded as one of the most difficult and crucial tasks for QSAR/QSPR modeling [1, 2].

There are several families of methods for addressing the descriptor selection problem, but most of them can be classified into two main categories: classic methods, such as those using multiple linear regression [3] or VSMP [4], and those using artificial intelligence-based techniques, such as the ones inspired on genetic algorithms, artificial neural networks or fuzzy logic methods [5–7]. In particular, the artificial intelligence-based methods are better suited to discover strong nonlinearities between the set of descriptors and a given biological activity (or property) and can overcome some limitations of classic descriptor selection methods [8].

Despite the numerous statistical approaches for feature or descriptor selection, real users that want to extract the most informative descriptors to understand or predict a property/activity still face an important challenge. The main reason is because none of these methods can be claimed as the best approach for any possible combination of dataset and prediction method. Therefore, in a real setting, especially when unseen data is likely to be presented in a future, users do not have a certain way of knowing what descriptor selection method would work best.

Another common criticism that descriptor selection methods receive is that most of these approaches are deemed as “black-boxes” by chemists. This is due to the fact that in order to fine-tune the results or introduce domain knowledge in the selection criteria it is necessary to know the inner workings of the method [9] or, in the best case, to encode this knowledge in the form of some prior probabilities. While some works have overcome this limitation by *ad*-*hoc* analyses [10, 11], we claim that there is a need for better support in the descriptor selection task, so that these methods can be embraced by the cheminformatics community.

There are two research questions that we aim at answering in this paper. The first one is whether we can leverage outcomes from different descriptor selection approaches to arrive at a more thorough decision on the subset of descriptors to be selected. While there exist several methods based on ensembles and voting schemes [12, 13] these approaches leave the chemical knowledge out of the selection process. The second research question is how we can involve the domain expert (e.g. a chemist), so that he or she can incorporate his or her knowledge and expertise during the feature selection process in a semi-automated manner.

In this paper we propose to deal with these two challenges by means of the proposal of a tool that combines statistical methods with interactive visualizations. This type of approaches falls under the emerging area of visual analytics [14]. The main idea behind visual analytics approaches is to merge the computational capacity of statistical and machine learning methods with the human natural ability of identifying patterns in visualizations. By allowing some form of interaction in the visualizations, domain experts can explore the data and provide feedback to the tool, and/or use the tool to arrive at more informative decisions. Visual analytics have been applied to different bioinformatics problems [15–17], while its application on cheminformatics has not been considered until recently. In the area of cheminformatics some approaches have been presented, such as CheS-Mapper [18], ChemMine [19] or MQN-Mapplet [20], which aim at interactively exploring chemical datasets by means of clustering and dimensionality reduction methods. QSARINS [21] is a comprehensive tool for multiple linear regression QSAR models that aggregates different statistical methods with a special focus on the model validation. INFUSE [22] is a recent visual analytics tool designed to help the analyst understand the predictive power of features in predictive modeling. One limitation of this approach is that the visual analysis is done on a per-feature basis. In contrast to the previous methods, in this tool we propose an approach that focuses on the challenging task of analyzing and selecting subsets of descriptors for QSAR/QSPR. This is done by means of explorative and visual analytics techniques, where the tool facilitates the comparison of multiple descriptors and descriptor subsets coming from the output of different descriptor selection methods.

## Implementation

As it was mentioned previously, when a physical chemistry expert develops a QSAR/QSPR predictive model, the choice of the most appropriate descriptors for this model constitutes the first complex challenge. Once the molecular descriptors have been computed for the dataset, different combinations of them should be analyzed in order to obtain a good quality model. In this context, the QSAR/QSPR model must satisfy two quality standards: high prediction accuracy, preferably evaluated with an external dataset, and good interpretability, evaluated from a physicochemical point of view. Considering the high number of molecular descriptors that are usually computed for a dataset, which could be in the range of thousands, a common practice consists of exploring different feature selection methods in order to obtain different smaller subsets of relevant descriptors [1, 5].

In a next step, these subsets should be systematically compared to find the best combination of descriptors. This process encompasses several aspects, such as analyzing descriptor co-occurrence in the different candidate models, avoiding redundant descriptor sets, and analyzing descriptor–target relationships. This analysis has been traditionally carried out by the expert combining her expertise with the design of plots and tables in an *ad*-*hoc* manner [10, 11].

This task can be tedious and time inefficient, because the relationships among the different pieces of information does not emerge in a straightforward way. At this point, it becomes valuable the contribution that visual analytics techniques can incorporate to this process. The main idea behind these strategies is to summarize and integrate data in a graphical and interactive way, facilitating the finding of key information for supporting domain expert decision making [14].

At this stage an interactive visual analytics tool, which we will refer to as Visual and Interactive DEscriptor ANalysis (VIDEAN), is proposed for comparing alternative descriptor subsets. These descriptor subsets are obtained from different descriptor selection methods as candidates for the QSAR/QSPR model. The main idea is to use this tool as a decision support system. Undoubtedly, the analyst’s task is complex since multiple aspects are involved in the modeling decisions. Therefore, different data exploration strategies are required in order to integrate these multiple pieces of information.

In this tool, we propose to visualize different aspects related to the information required for modeling, uncovering hidden connections among the descriptors and their relationships with the target property. In this way, the specific objectives that guided the design of these visualizations can be defined as follows:

1. The avoidance of redundant descriptors in QSAR/QSPR models. This means that if two or more descriptors are conveying similar information, the tool should help choose the most meaningful or suitable descriptors for the model, and thus keeping the descriptor subset small.
2. The complementarities among the selected descriptors and the target property. This means that each descriptor of the model should be relevant for prediction. In other words, if one descriptor were removed, the prediction accuracy of the model should worsen.

### Interactive visualizations proposed for QSAR/QSPR modeling

**Fig. 1:**
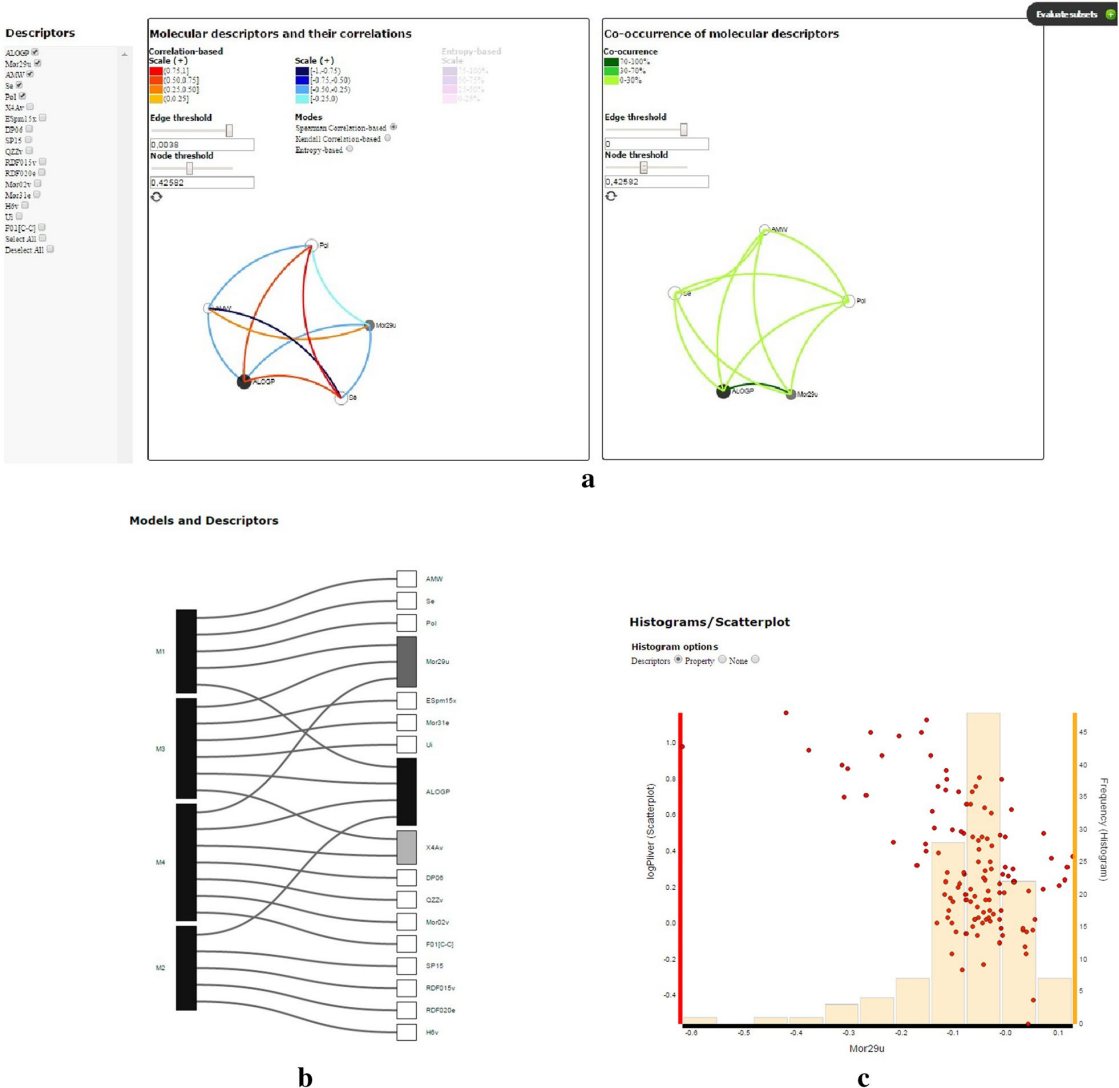
**a** Primary (*left*) and secondary (*right*) undirected graphs (G_p_ and G_s_, respectively), which focus on pairwise associations between descriptors. **b** Bipartite graph that represents the molecular descriptors grouped in each model. **c** *Scatterplot* and *histograms* for showing the relationship between descriptors and the target property.

In this section, the visualizations included in VIDEAN are introduced. Some examples of the most representative decisions that a modeler can take from these visualizations are also illustrated. In VIDEAN, the interface is organized around the four charts depicted in Fig. 1. The upper section of the screen contains two undirected graphs that represent pairwise associations between descriptors. The lower section of the screen contains a bipartite graph, which represents the relationship among the candidate subsets of descriptors (called “models” in this tool^a^) and individual descriptors, and an interactive plot area, which shows different relationships between the descriptors and the target property. In this way, the modeler can analyze multiple aspects involved in the descriptor selection process simultaneously.

#### Undirected graphs for pairwise descriptor analysis

In these charts the information is represented as undirected graphs, where each node (circle) represents a descriptor selected by at least one of the models (Fig. 1a). The graph on the left has a central role in the analysis and it will called primary undirected graph (G_p_), whereas the graph on the right plays a complementary role and it will called secondary undirected graph (G_s_). In both graphs, the node color uses a grayscale to indicate the proportion of models in which the descriptor has been selected: white, if it was chosen only by a single model, and black, if it was chosen by all the models. In this way, the consensus among the different models is incorporated in the analysis [23].

In G_p_, the node sizes and edge weights can be customized for representing different types of relationships among descriptors. The two main modes are entropy-based and correlation-based. In the first mode, node sizes are associated with the conditional entropy of the descriptor with respect to the target property. A smaller node size indicates a higher value of conditional entropy and a bigger node size indicates a lower value of conditional entropy. Conditional entropy is a measure of the amount of uncertainty over a random variable when the value of another random variable is known. In this case, it is a measure of how much uncertainty remains about the target property value when we know the value of the descriptor. In this mode, edges represent the mutual information between descriptors. Mutual information (MI) measures the amount of information that a random variable contains about another. Thus, the mutual information between two descriptors is the reduction in the uncertainty of one of them due to the knowledge of other, and vice versa [24]. Note that MI is a metric, so the value obtained from this calculation will always be non-negative and symmetric [25]. Edge color is used to quantify edge weights, and it ranges from a pink to violet scale that indicates the MI value between two descriptors. Light pink is used when the descriptors are independent (MI = 0). The opposite case, dark violet is used when the descriptors are identical (high MI values) i.e. the information derived from one of them can be used to represent the other one.

In the second mode, the node sizes are associated with *Spearman´s rank correlation* (or *Kendall*) between descriptors and its target property, whereas the edges represent *Spearman´s rank correlation* (or *Kendall*) between descriptors. Here, a smaller node size indicates a lower correlation and a bigger node size indicates a higher correlation with the target property. The edge color range goes: from red to yellow for positive correlation (red = 1) and from blue to light blue for negative correlation (blue = −1). Spearman and Kendall correlations, as opposed to the well-known Pearson, allow identifying non-linear relationships. In G_s_ (Fig. 1a, graph on the right), the node features (size and color) are automatically fixed by the mode selected for G_p_. In this graph the edges represent the degree of co-occurrence of the descriptors in the models. In other words, the co-occurrence of two descriptors is computed using the following ratio: the number of models in which both descriptors appear simultaneously over the total number of models. The range of colors used for the edges ranges from dark green (high co-occurrence) to light green (low co-occurrence). In this graph the modeler may recognize pairs of relevant co-occurring descriptors, namely those with high importance for the prediction of the property as a pair (not only individually). These situations could be related to complementary physical–chemical information between two descriptors. For example, molecular weight and polarizability together can be useful for predicting a partition property between two solvents, although the individual contribution of these descriptors may not be so relevant.

#### Bipartite graph for model vs. descriptor analysis

In this chart the information is represented by a bipartite graph, where all nodes are represented by boxes. The nodes on the left represent the models and the nodes on the right represent the descriptors of these models. The edges indicate occurrence of a descriptor in a model. In this way, we can have an overview of the models under study (Fig. 1b). It is important to clarify that a QSAR model is constituted by a subset of descriptors and the relationship that associates these descriptors with a target property. However, in this paper a “model” makes reference to a candidate subset of descriptors only. Therefore, the use of the word “model” is frequently used here as a simplification of “candidate subset of descriptors”.

#### Additional plots for descriptor vs. property analysis

An extra feature associated with the graphs consists on showing the distribution of descriptor values with respect to the target property using additional visualizations (Fig. 1c). Clicking on a G_s_ node shows a scatterplot with the dispersion of the descriptor values versus the target property values for all compounds in the dataset. Additionally, two histograms indicating frequency of descriptor and target values can be overlapped over the scatterplot. At this point, the modeler can have a better understanding of the contribution of each descriptor to the modeling of the target property and the type of relationship (linear, quadratic, cubic, etc.). For example, in previous works [11, 26], the best models were obtained by combining descriptors that cover different subregions of the chemical domain. In other words, the exploration of this visualization allows assessing the contribution of the different descriptors to the model.

#### List of descriptors

This list shows all the descriptors involved in the analysis. This list has an important role as it controls what can be visualized by the graphs Gp and Gs. The selection (unselection) of a descriptor from the list involves the addition (removal) of the corresponding node on G_p_ and G_s_. Furthermore, the selected descriptors in the list can also be changed from the visible nodes on G_p_ or G_s_.

#### Prediction models

VIDEAN allows modeling the target property using different statistical methods. The descriptors that are used to build the model are those selected in the list of descriptors. Currently, there are three available methods, namely, linear regressions, decision trees and neural networks, which can be parameterized accordingly. These prediction methods are implemented using WEKA libraries [27]. While the focus of VIDEAN is not on finding the best predictive model, this feature allows the comparison of the predictive capacity of several subsets of descriptors using some baseline methods.

#### Interactions with the visualizations

The undirected graphs allow several interactions with the user. Initially, the graphs are reduced on their nodes and edges based on different thresholds. When the node (edge) threshold slider or value is modified, the graph is updated according to these values.

When hovering over a node, the nodes and links connected to it are highlighted and the others are fainted leaving a clear contrast without losing the global context. When hovering over an edge, the numeric value encoded by the color is shown. This number can represent an MI value, a correlation, or the number of models depending on the graph and edge coding mode.

By double-clicking over a color scale, the links within this color range are shown and everything else is fainted. The list with the definition of each descriptor can be brought up by double-clicking on a node. Note that by using these interactions, a modeler could discover interesting descriptors. For example, non-redundant descriptors, selected in most of the models and strongly correlated with the target property can be easily identified in G_p_ by detecting the bigger and darker nodes connected between each other with light-colored links.

Finally, the interactions are slightly different for the bipartite graph. When hovering over a model node, the edges that connect this model with its descriptors are highlighted while the other descriptors are fainted. When hovering over a descriptor node, the edges to the models in which the descriptor is present are highlighted, while the remaining edges are fainted. Note that the colors used for the descriptor nodes (scale from white to black) are the same in the three charts based on graphs. Also, by clicking on a model-type node, their descriptors are updated on the main list, and hence also on both graphs. The modeler can use this visualization to better understand differences among models, and to create new models from their combination. Thereby, it is possible to achieve new models combining descriptors from different models, as it will be illustrated in next sections.

## Results and discussion

In this section, the applicability of the proposed tool is studied in two different scenarios. The first scenario corresponds to the prediction of log P_liver_, which is a relevant property for the study of *volatile organic compounds* (VOCs). In this case the tool is used to choose one subset of descriptors from a group of candidate subsets obtained automatically by descriptor selection methods. The second scenario is related to the prediction of a mechanical property that plays a central role in the design of polymers. In this case, the tool is applied in a different strategy, where an expert wants to combine his domain knowledge with the automatic selections reported by some descriptor selection methods.

### Log P_liver_ analysis

Volatile organic compounds (VOCs) are emitted as gases from some solids or liquids. VOCs include a variety of chemicals and many of them have adverse health effects. Several methodologies have been applied to VOCs inhalation studies [28, 29] and are related to the analysis of physiologically-based pharmacokinetic (PBPK) models. PBPK modeling is a mathematical modeling technique for predicting the impact of synthetic or natural chemical substances in humans and other animal species. In respiratory PBPK models blood–air, liver–air and liver–blood partition coefficients of VOCs are important for their hazard assessment and bioavailability estimation [30]. Several attempts have been made to model the relationship between the structure or molecular properties and the blood-to-liver distribution, usually denoted as log P_liver_, of VOCs and drugs [11, 30].

The objective of the use case reported here is to choose a subset of descriptors to predict log P_liver_ in such a way that the model shows high accuracy and it is also interpretable in terms of its physicochemical descriptors. Particularly, in this case we aim at choosing one out of four QSAR models generated automatically by a descriptor selection tool [5], using a dataset of 122 VOCs and 1,391 molecular descriptors. The compounds were extracted from [11] consisting of hydrocarbons, alkyl halides, alcohols, ethers, esters, ketones, epoxides, nitriles, halobenzenes, polycyclic hydrocarbons and benzene derivatives. The descriptors involved in each of the four models are listed in Table 1. All these models have similar predictive capabilities in terms of mean error and coefficient of determination as it can be seen in the second column of Table 1.

**Table 1:** Candidate models obtained for log P_liver_ using the dataset reported by [11]

Model	Predictive accuracy	Subset cardinality	#Frequent descriptors	#Descriptors shared with other model
M1 (ALOGP, Mor29u, AMW, Se, Pol)	R^2^ = 0.81 MAE = 0.15 RMSE = 0.20	5	2	2
M2 (ALOGP, SP15, RDF015v, RDF020e, H6v)	R^2^ = 0.76 MAE = 0.17 RMSE = 0.23	5	1	1
M3 (ALOGP, Mor29u, X4Av, ESpm15,Mor31e, Ui)	R^2^ = 0.79 MAE = 0.16 RMSE = 0.21	6	3	3
M4 (ALOGP, Mor29u, X4Av, DP06, QZZv, Mor02v, F01[C–C])	R^2^ = 0.79 MAE = 0.16 RMSE = 0.21	7	3	3

The second column shows the predictive accuracy of the “best” model after applying 4-fold cross validation on three different methods (linear regression, decision trees, and neural networks). In this case, the best predictive accuracy for the four models was obtained by using a decision tree (M5P). The parameter setup and predictive accuracy for all methods are available in the Additional file 1: Table S1.

#### Choosing the best subset of descriptors

When subsets of descriptors with similar predictive capabilities are compared, the choice of a subset usually focuses on those with lower cardinality. This is because a subset that involves a smaller number of descriptors is generally easier to interpret and more likely to be generalizable (based on the Ockham’s razor principle [31]). Despite this, and given that the differences between the cardinalities of the candidate subsets are minimal, other criteria, such as interpretability or confidence, are taken into account.

**Fig. 2:**
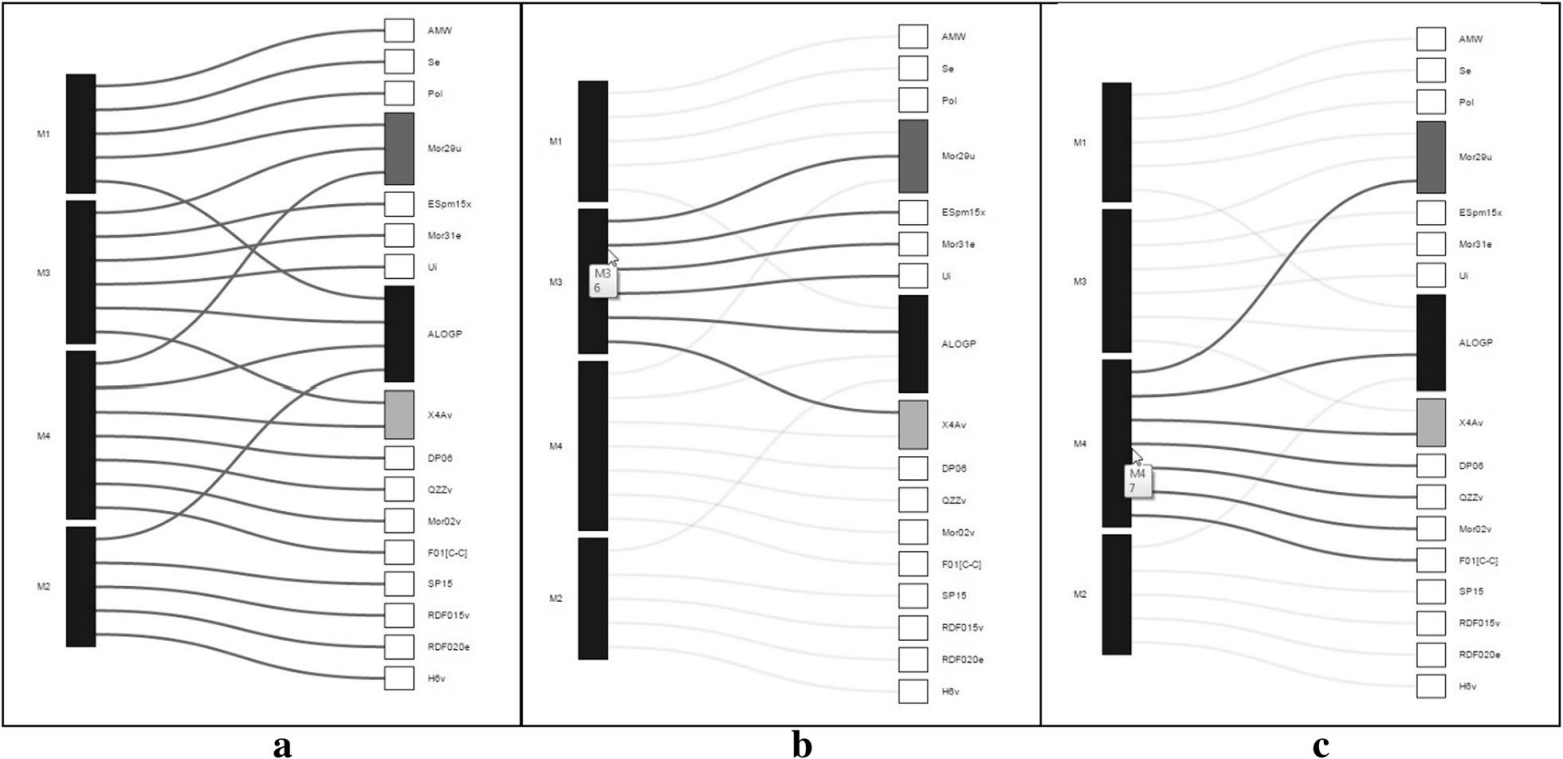
**a** Relationships among models and descriptors. Frequent descriptors correspond to nodes that are filled with a darker *gray* color. **b** Visualization when hovering over M3. **c** Visualization when hovering over M4.

A first strategy can be to analyze the subsets that contain a higher proportion of “frequent descriptors”, which are those that are present in more than one candidate subset. The rationale that motivates us to start the analysis exploring these descriptors is that their occurrence in more than one subset would indicate a higher likelihood of contributing relevant information for modeling the property. This information can be easily visualized by interacting with the bipartite graph of models and descriptors. Figure 2 shows that M3 and M4 are the models that contain more frequent descriptors (three), but also higher cardinality. In this way, if we consider cardinality and number of frequent descriptors, we can conclude that M2 would be the least valuable.

**Fig. 3:**
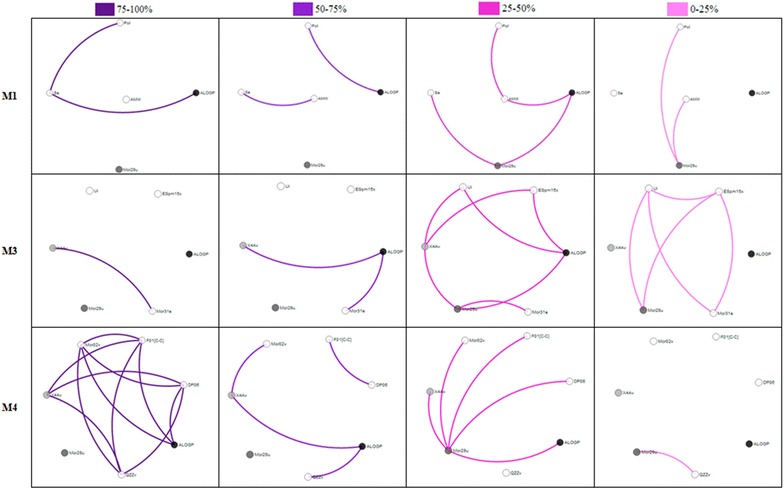
Links with the four levels of mutual information (*columns*) between the descriptors of M1, M3 and M4 (*rows*). This filtering can be obtained by following these steps: (1º) select each model by clicking on the corresponding node on the bipartite graph; (2º) having selected the entropy-based mode, move the edge threshold on the G_p_ to the right until it stops; (3º) filter edges by double-clicking over the color range above the graph.

Once M2 is discarded, a next step can consist of analyzing mutual information between the descriptors that are present in the remaining models. The objective here is to determine which models have a higher proportion of descriptors that contribute non-redundant information for the prediction (modeling) of the property. Figure 3 shows this information for M1, M3 and M4. It can be clearly seen that M4 has higher pairwise mutual information between their descriptors, and thus it will be regarded as the least interesting model due to the high redundancy among their descriptors.

**Fig. 4:**
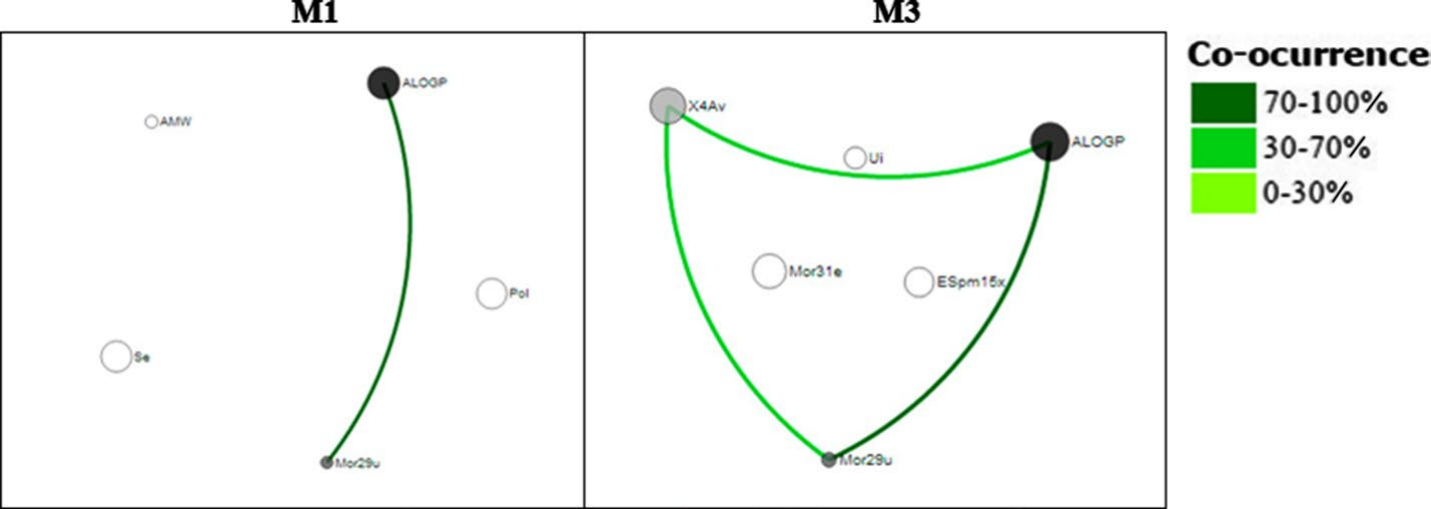
Medium and high degree of co-occurrence between descriptors of M1 and M3. This filtering can be obtained by selecting each model by clicking on the corresponding node on the bipartite graph, and then modifying the edge threshold on G_s_.

After M4 is discarded, the following analysis focuses on deciding between M1 and M3 using the remaining visualizations. A first step consists in assessing descriptor co-occurrence in these models. This is in order to identify similarities and discrepancies among descriptors of different models. This information is shown in Fig. 4, where it can be observed a similar proportion of descriptors with high and medium degree of co-occurrence.

At this point, the histograms and scatterplots can be used for analyzing which one of the two models is more interpretable from a physicochemical point of view. M1 consists of five descriptors: AMW (molecular weight divided by the number of atoms), Se (sum of atomic Sanderson electronegativities), Pol (Polarity number), Mor29u (3D-Molecule Representation of Structures Based on Electron diffraction—signal 29/unweighted) and ALOGP (Ghose–Crippen octanol–water partition coefficient). All of them provide to a lesser or greater extent important information about molecular properties related to the molecule capability to distribute between the two media under study: liver tissue and blood. Liver tissue may be considered as a non-polar medium while blood is a polar one. The descriptor AMW discriminates the molecules taking into account their atomic composition (type and quantity). Plotting the compounds by AMW values allows identifying different chemical families, where differences in their physicochemical properties become more evident, e.g. differences in their polarity properties. Something similar happens to the descriptor Se which succeeds in discriminating the VOCs families with the sum of Sanderson atomic electronegativities (scaled on carbon atom). The descriptors Pol and Mor29u highlight structural 2D and 3D properties, respectively. Pol relates to the steric properties of molecules and it works as a specific filter that discriminates molecules by chain length. Mor29u captures minimum variations in 3D-structural features based on interatomic distances, and it can differentiate isomeric structures but it does not differentiate chemical families. Finally, ALOGP gives relevant information about molecular affinity for an octanol–water medium, and it is sensitive to minimum differences in molecular structure. There is certain correlation between this descriptor and log P_liver_, because polar molecules have low ALOGP and log P_liver_ values (e.g., alcohols and halogenated hydrocarbons) while non-polar ones have high values (e.g., alkanes and aromatics). All variables were shown to be relevant to the performance of the model confirming that the contribution is global. In this sense, an expert analysis would determine that descriptors of M1 contemplate more physicochemical aspects related with the property under study. This fact can be observed from the histograms and scatterplots, where all distributions of variables are different as it can be seen in [11]. Furthermore, M1 also has a lower cardinality than M3.

In this way, we have shown a possible flow of analysis to choose between alternative models using our visual analytics tools. Nevertheless, it is important to emphasize that this strategy is not the only approach to follow for the analysis. For example, a user could start analyzing the interactions between the descriptors of the models through the inspection of undirected graphs. While different flows of analysis can lead to different descriptor subsets, an important aspect of this type of selection is that it increases the expert’s confidence on the model.

### Elongation at break analysis

In the polymer industry it is fundamental to define the profile of applicability of a polymeric material. There are numerous mechanical properties that can describe this profile, and among them, the capacity to resist breaking under tensile stress is one of the most important and widely measured material properties used in structural applications [32]. In this example, a property obtained from a tensile test is explored: elongation at break, which is a measure of material ductility. It is important to consider that not only the ambient temperature affects the final value of elongation at break, but the rate (crosshead speed) also does [33]. Values of elongation at break reported at specific temperatures and cross-head speeds are typical for these test conditions. A dataset of high molecular weight polymers extracted from [34] was used, which includes 655 molecular descriptors coming from 77 samples of neat resins, amorphous, linear, non-cross-linked, and non-elastomer polymers.

The purpose of this example is to predict elongation at break by means of a model with high performance, interpretability and the special requirement of including CHS (crosshead speed) as an experimental descriptor because of its high influence on the property value. Note that elongation at break values of this dataset were obtained at different rates. In this case, the analysis started from the best ten subsets automatically obtained by the descriptor selection method proposed by [5]. Their prediction accuracy and cardinality are shown in Table 2.

**Table 2:** Prediction accuracy and cardinality for the best ten models obtained by Soto’s method [5]

Model	Predictive accuracy	Cardinality
M1 (Mn/MW, Sp, RHyDp, ETA_EtaP_F_L)	R^2^ = 0.26 MAE = 4.62 RMSE = 8.14	4
M2 (Mn/MW, MDEO-11, D/Dr09, SMTIV)	R^2^ = 0.32 MAE = 5.94 RMSE = 8.31	4
M3 (Mn/MW, nHBint4, nHBint10, ETA_dEpsilon_B)	R^2^ = 0.56 MAE = 4.03 RMSE = 6.22	4
M4 (Mn/MW, nsCH3, nF6Ring, ALOGP2, RDCHI)	R^2^ = 0.41 MAE = 3.94 RMSE = 6.75	5
M5 (Mn/MW, nROH, n6Ring, nHCsatu, ALOGP2)	R^2^ = 0.68 MAE = 3.28 RMSE = 5.78	5
M6 (Mn/MW,nP, minHBa, T(O..P), ETA_Epsilon_3)	R^2^ = 0.25 MAE = 4.48 RMSE = 7.20	5
M7 (Mn/MW, ETA_dEpsilon_B, C-005, SHaaCH, nHBint9,nCt)	R^2^ = 0.31 MAE = 4.19 RMSE = 7.20	6
M8 (Mn/MW, ndssC, minHBint9, MSD, C-004, Mw/Mn (PDI), crosshead speed(CHS))	R^2^ = 0.39 MAE = 3.92 RMSE = 6.86	7
M9 (Mn/MW, Pol, Wap, maxHAvin, nHAvin, MWC04)	R^2^ = 0.15 MAE = 4.92 RMSE = 7.88	6
M10 (Mn/MW,maxHBint6, ETA_dEpsilon_A, TIC2, ndO, nHdCH2)	R^2^ = 0.48 MAE = 4.02 RMSE = 7.09	6

The second column shows the predictive accuracy of the “best” model after applying 4-fold cross validation on three different methods (linear regression, decision trees, and neural networks). The parameter setup and predictive accuracy for all methods is available in the Additional file 1: Table S2.

### Designing a new model incorporating CHS

**Fig. 5:**
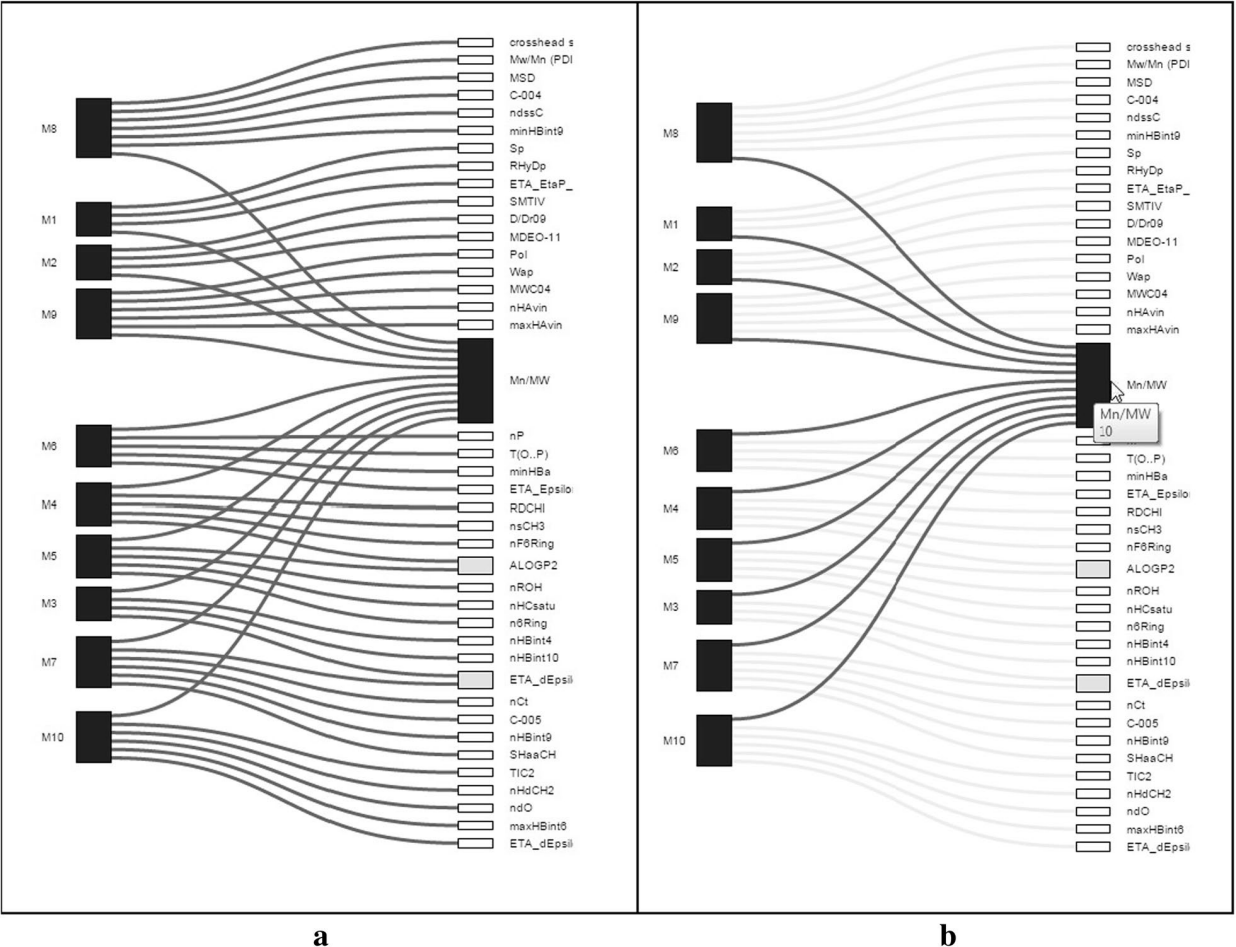
**a** Membership relation between models and descriptors. Frequent descriptors correspond to nodes that are filled in shades of *gray*. **b** View after hovering over the most frequent descriptor.

As a first strategy the “frequent descriptors”, i.e. those that are colored in a darker grayscale and indicate that they have been chosen by more than one model, were explored. Figure 5 shows that Mn/MW (in black) was chosen by all models, and ETA_dEpsilon_B as well as ALOGP2 were selected in two models each (in gray).

Then, the modeler is faced with the analysis of whether these descriptors provide valuable information to describe the target property from a physicochemical point of view. This deserves a brief discussion. Mn/MW is a descriptor relating the number-average molecular weight (Mn) to the molecular weight of the monomer of the polymer, giving as a result the number of repeating units in an average polymer chain. In other words, Mn/MW provides *macro* information of the material. ETA_dEpsilon_B is an extended topochemical atom descriptor, which represents a measure of contribution of unsaturation and it is calculated on the monomer. ALOGP2 is the square of ALogP (Ghose-CrippenLogKow). These partition coefficients represent a measure of hydrophobicity of the monomer. ETA_dEpsilon_B and ALOGP2 give information at a *micro* level because they were calculated on a monomer and not on an average polymer molecule (impossible to compute). Nevertheless, this physicochemical information is related to flexibility and intermolecular forces of the material. Therefore, the “frequent descriptors” are representing *macro* and *micro* aspects of the dataset molecules and all of them affect elongation at break.

**Fig. 6:**
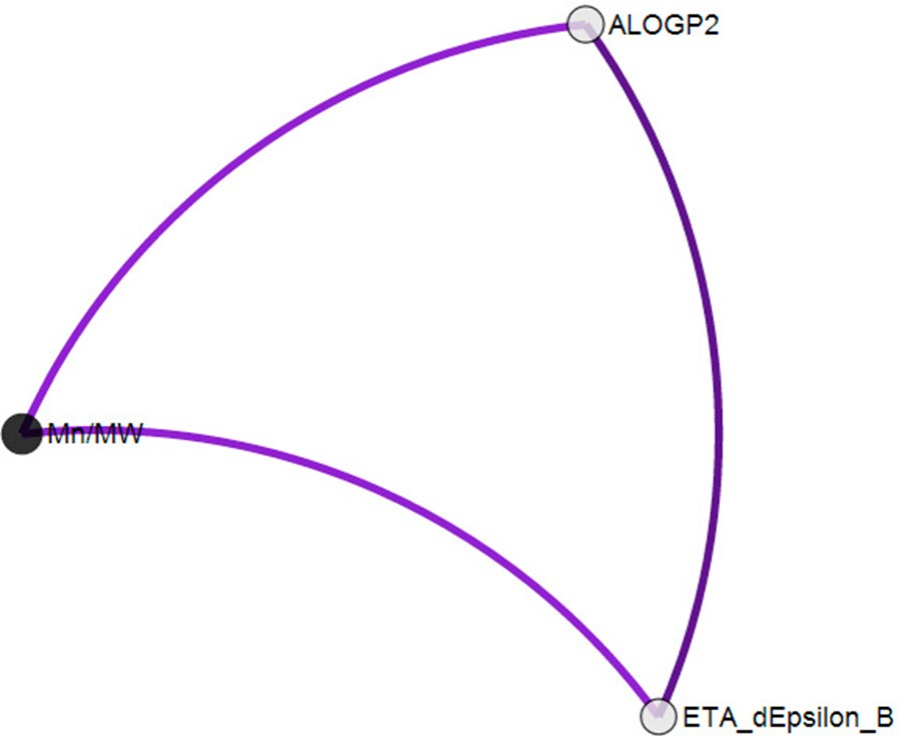
Mutual information among the three most frequent descriptors: Mn/MW shows greater independence compared to the other two descriptors, but the opposite occurs between ETA_dEpsilon_B and ALOGP2. This filtering can be obtained by following these steps: (1º) having selected the entropy-based mode, check the option “Deselect All” on the list of descriptors; (2º) select from the list these three descriptors.

One possible question for the modeler could be: how independent from each other the information provided by each descriptor is? Mutual information (Fig. 6) was then analyzed aiming at building a model whose descriptors are independent in terms of the numerical information they provide, thus avoiding redundancy.

**Fig. 7:**
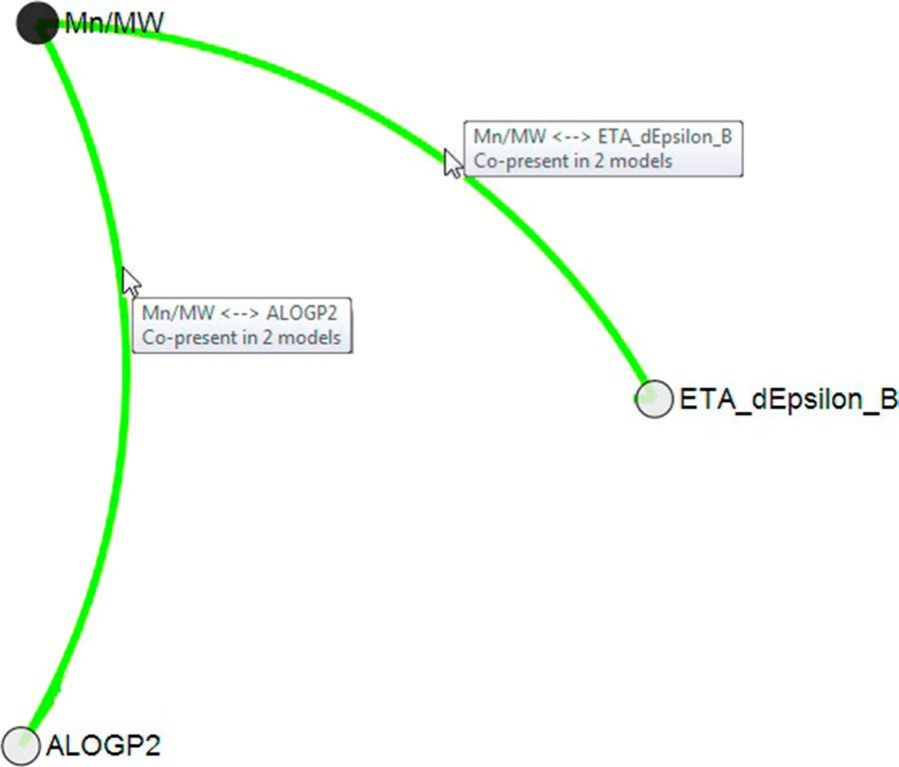
Co-occurrence degree among frequent descriptors. This filtering can be obtained using the same steps described in Fig. 6 and then modifying the edge threshold.

The co-occurrence of these “frequent descriptors” was also analyzed to find some complementary information among them. As it is shown in Fig. 7, two pairs appear twice: Mn/MW-ETA_dEpsilon_BandMn/MW-ALOGP2, but ETA_ dEpsilon_B and ALOGP2 do not co-occur.

**Fig. 8:**
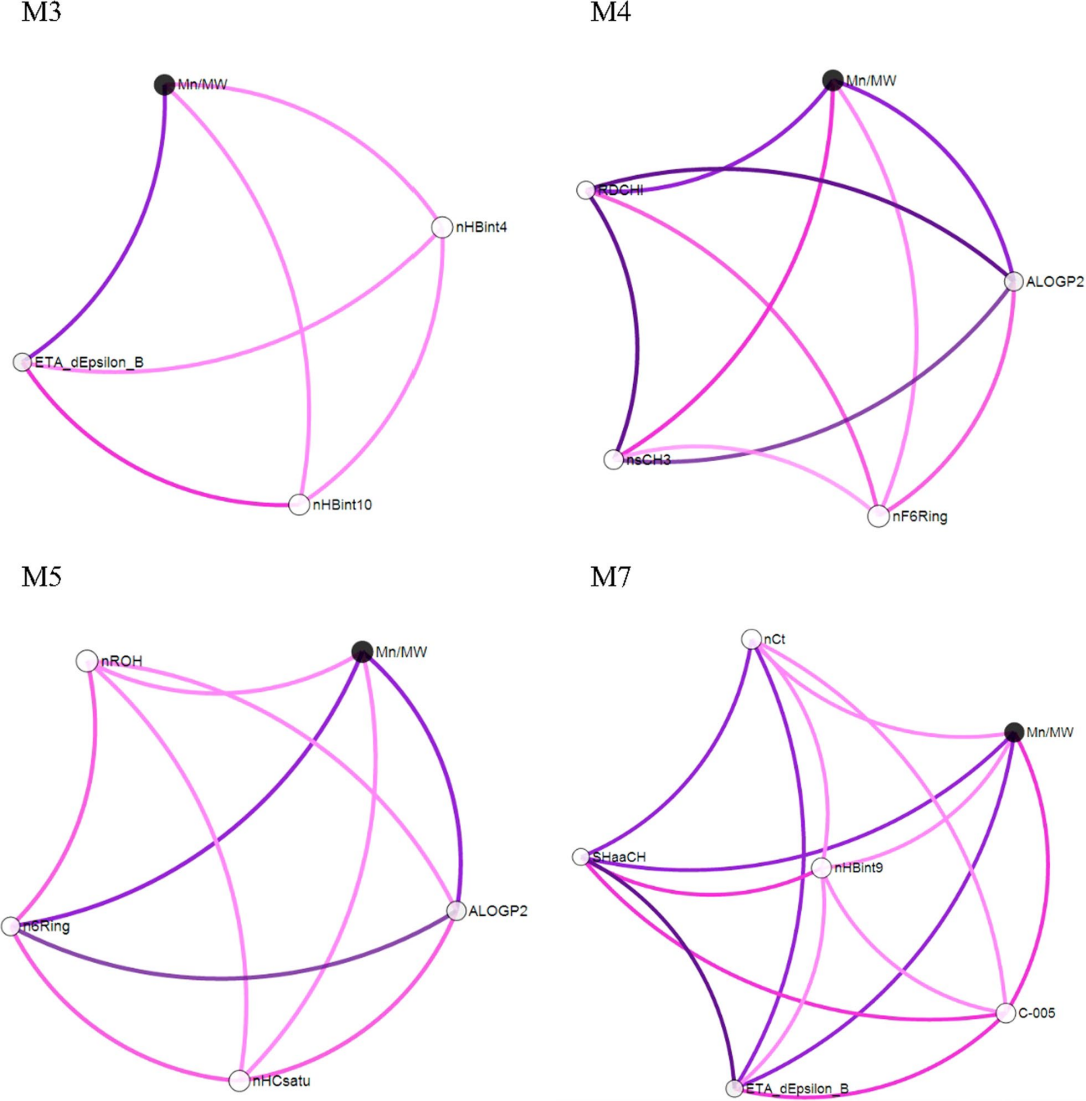
Mutual information (high, medium and low) between descriptors of Models 3, 4, 5 and 7. This filtering can be obtained by following these steps: (1º) select each model by clicking on the corresponding node on the bipartite graph; (2º) having selected the entropy-based mode, move the edge threshold on the G_p_ to the right until it stops.

After this first stage of analysis, the four models containing “frequent descriptors” were chosen, i.e. M3, M4, M5 and M7 (Fig. 5), because they provide valuable information for the description of the property. The quality of the four selected subsets was assessed considering independence, cardinality and interpretability. In Fig. 8, it can be seen that the subset that best meets all these factors is M3. This subset consists of: Mn/MW, ETA_dEpsilon_B, nHBint4 and nHBint10. The latter two descriptors are electrotopological descriptors: count of E-State descriptors of strength for potential Hydrogen Bonds of path length 4 and 10, respectively, which represent structural and chemical aspects of monomers.

**Fig. 9:**
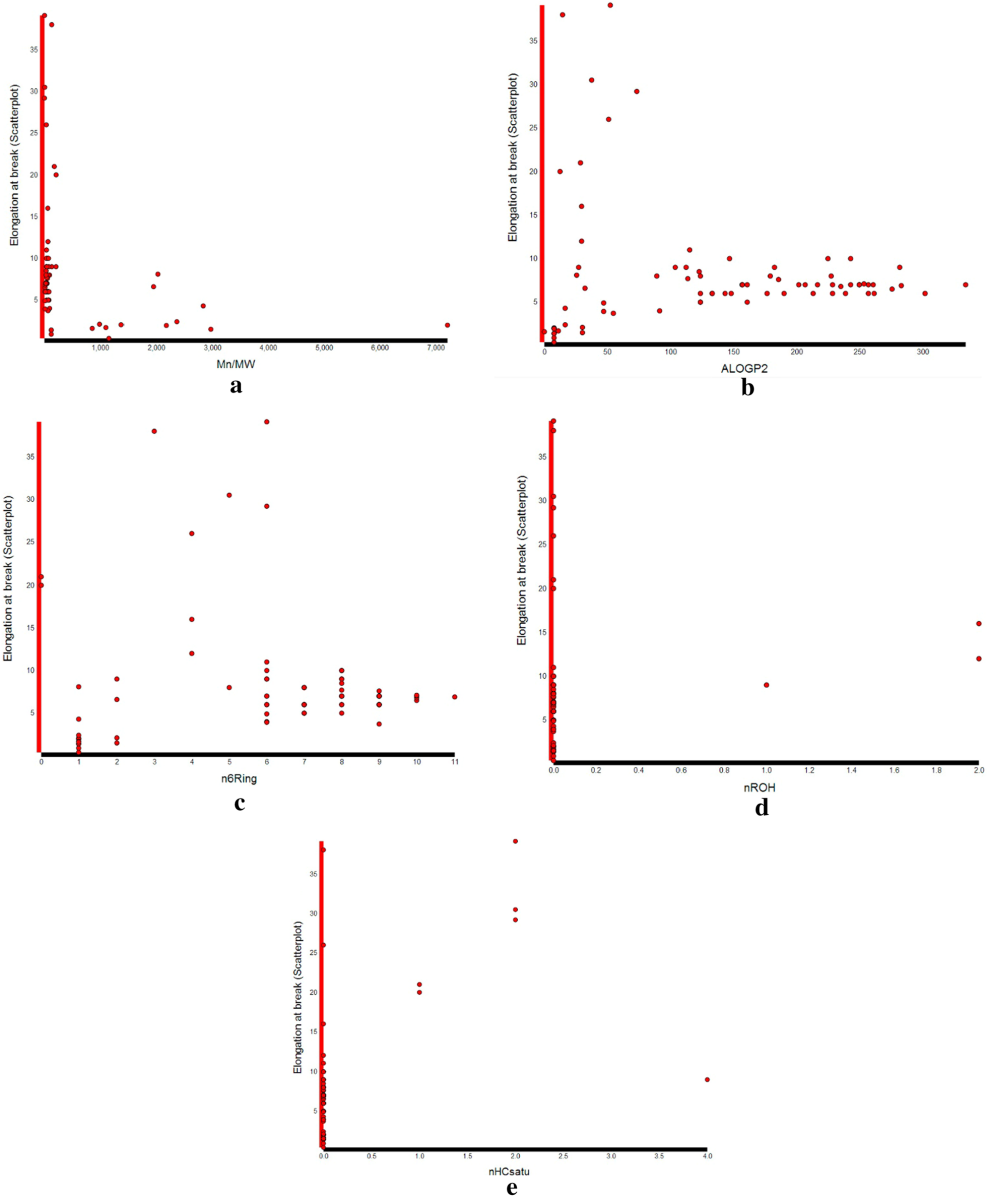
*Scatterplots* of descriptor values vs. *elongation at break* for M5. This can be obtained by clicking on the corresponding G_s_ node. Descriptors Mn/MW, ALOGP2, n6Ring, nROH and nHCsatu are plotted in each panel, respectively (**a**–**e**).

Considering the analysis from a predictive point of view, note that in Table 2 the best prediction accuracy belongs to M3 (R^2^ = 0.56) and M5 (R^2^ = 0.68). However M5 has greater cardinality (5) than M3 (4) and two of the descriptors of M5 (nROH and nHCsatu) almost do not va ry with respect to the target (Fig. 9d, e). This lack of variance is undesirable, since it means that the numerical information provided is very limited.

Subsequently, the modeler may want to modify M3 by adding additional descriptors. In this case, CHS is considered essential to describe the property, as the dataset values for elongation at break were measured at different crosshead speeds. The resulting subset contains five descriptors (M3 + CHS) and the next step is to analyze it statistically, in order to check whether the modeler hypothesis improves the accuracy of prediction. In Table 3, it can be seen that the statistical values confirm the expert’s suggestion, and consequently the new model (R^2^: 0.62) outperforms the original one (R^2^: 0.56).

**Table 3:** Predictive accuracy of the models M3, (M3 + CHS) and (M3 + CHS − nHBint4)

Model	Predictive accuracy
M3 (Mn/MW, nHBint4, nHBint10, ETA_dEpsilon_B)	R^2^ = 0.56 MAE = 4.03 RMSE = 6.22
M3 + CHS (Mn/MW, nHBint4, nHBint10, ETA_dEpsilon_B,CHS)	R^2^ = 0.62 MAE = 3.43 RMSE = 5.89
M3 + CHS − nHBint4 (Mn/MW, nHBint10, ETA_dEpsilon_B,CHS)	R^2^ = 0.69 MAE = 3.24 RMSE = 5.68

The second column shows the predictive accuracy of the “best” model after applying 4-fold cross validation on three different methods (linear regression, decision trees, and neural networks). In this case, the best predictive accuracy for the three models was obtained by using a decision tree (M5P) and evaluating using 4-fold cross validation. The parameter setup and predictive accuracy for all methods is available in the Additional file 1: Table S3.

**Fig. 10:**
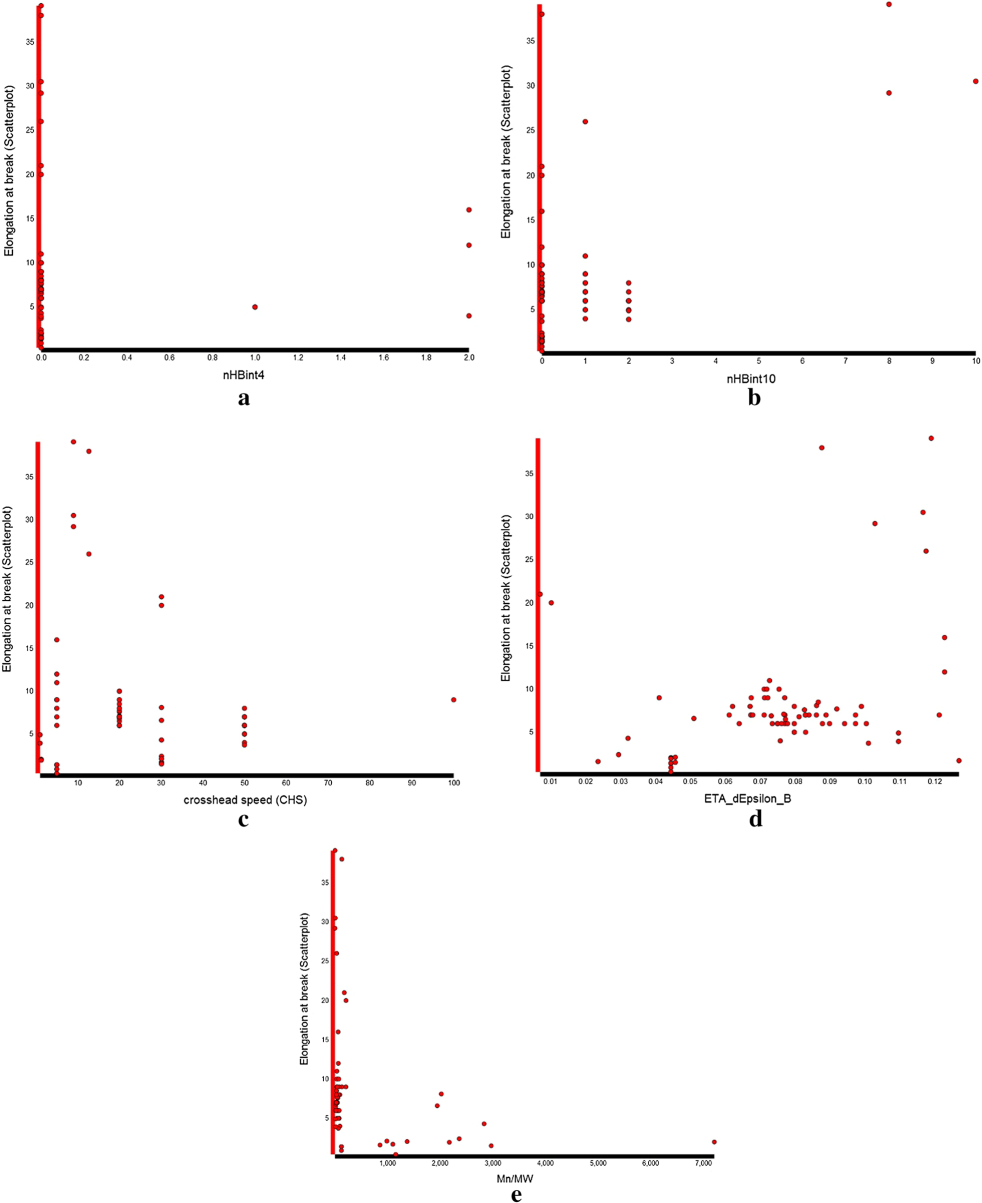
*Scatterplots* of descriptor values vs. elongation at break for M3 + CHS. Descriptors nHBint4, nHBint10, crosshead speed (CHS), ETA_dEpsilon_B and Mn/MW are plotted in each panel, respectively (**a**–**e**).

In order to further enhance the study, scatterplots with the variation of descriptor values with respect to elongation at break (Fig. 10) were analyzed. At a first glance, the lack of variation of nHBint4 descriptor is noteworthy (Fig. 10a). This behavior motivated the removal of NHBint4 from the model. In Table 3, it can be seen that the predictive accuracy of the new model improved (R^2^: 0.69). Additionally, from the remaining subplots (Fig. 10b–e), it is clear that all descriptors vary differently with the property which is another indication that they are likely to provide independent information.

To sum up, this example illustrates a possible flow of analysis to generate a robust model for the prediction of elongation at break of polymers by using our visual tool. This analysis took into account statistical and physicochemical aspects, allowing the expert to modify and test the final model by considering a previous hypothesis. The final model (M3 + CHS—nHBint4) contains four descriptors that provide meaningful and independent information: Mn/MW, ETA_dEpsilon_B, nHBint10 and CHS. Mn/MW represents the number of repeating units of average polymer molecules, associated with the average chain length (*macro*), nHBint10 and ETA_dEpsilon_B represent *micro* level structural properties and finally CHS provides experimental information from the property measurement test, which affected the predictive capacity of the model markedly. Thus, the model considers molecular flexibility, intermolecular interactions and test rate, which makes it a reliable and interpretable subset.

## Conclusions

The selection of a subset of molecular descriptors during the design of a QSAR/QSPR model is a hard task, where several simultaneous goals must be satisfied [1, 35]. Many feature selection methods used for dealing with this problem are focused on statistical relationships among the descriptors and target properties, leaving aspects associated with the chemical knowledge out of the picture. Therefore, the interpretability and generality of the models obtained by these methods are drastically affected. For this reason, a strategy that incorporated expert knowledge in the selection process is required in order to improve the user confidence in the final set of descriptors.

In this paper we address this challenge by proposing a software tool that conjoins statistical methods with interactive visualizations. The main idea is to exploit the expertise of chemists by an interactive visual exploration of different pieces of data generated from statistical and information theory based metrics. Several coordinated visual representations were designed with the aim of capturing different aspects and relationships among descriptors and target properties. Some key insights that can be gained aided by the interactive visualizations are: redundant descriptors, descriptors that provide discriminative information, relevant descriptors by consensus among alternative models, and descriptors whose knowledge helps reduce the uncertainty about the value of the target property.

The capabilities of the proposed tool were assessed through two different scenarios. The first case study corresponds to the prediction of log P_liver_. In this experiment the tool was used to choose one subset of descriptors from a group of alternative subsets generated automatically by one or more descriptor selection methods. The second study corresponds to the prediction of a relevant mechanical property useful in the design of polymers. In this case, we illustrate the case where the analyst wants to make an intervention in the automatic selection of descriptors in order to incorporate an experimental descriptor to the model. Thus, we showed how the expert can use our tool to analyze information coming from existing descriptor subsets and even incorporate new descriptors according to her own knowledge of the target property.

The results in both cases showed the suitability and convenience of this methodology for selecting sets of descriptors with desirable characteristics (low cardinality, high interpretability, low redundancy and high statistical performance) in an exploratory and versatile way, and with a lower “cognitive effort” in comparison with the alternative of using an ad-hoc manual analysis of the selected descriptors.

Besides the aforementioned advantages of incorporating the analyst in the loop, it is also important to establish the limitations and scope of this software. Here we focus on the descriptor selection stage. In order to address the more general problem of activity/property prediction, we would need to include aspects such as studying the compounds individually or in families, as well as the incorporation of visualizations related to the applicability domain of the QSAR models or the analysis of its outliers.

Finally, this analysis can be repeated by using different prediction methods, which could lead to different results. Yet the focus of this use case is not in the prediction or validation of the results themselves but on a rationale set of steps to find good candidate descriptors. These descriptors should then be evaluated in an unbiased manner and preferably using an external dataset [10].

## Availability and requirements

Project name: Visual and Interactive DEscriptor ANalysis (VIDEAN)

Project home page: http://lidecc.cs.uns.edu.ar/VIDEAN/

Operating system(s): Platform independent

Programming language: Javascript and Matlab

Other requirements: Web browser (tested with Chrome)

License: BSD

Any restrictions to use by non-academics: None

Source code is available at GitHub: https://github.com/jimenamartinez/VIDEAN

## Endnote

^a^It is important to clarify that a QSAR model is constituted by a subset of descriptors and the relationship that associates these descriptors with a target property. However, in this paper a “model” makes reference to a candidate subset of descriptors only. Therefore, the use of the word “model” is frequently used here as a simplification of “candidate subset of descriptors”.

## Additional file

The following additional data are available with the online version of this paper.
Additional file 1. The parameter setup and tables with the predictive accuracy of models used in the case studies.

## References

[1] Goodarzi M, Dejaegher B, Heyden YV (2012). Feature selection methods in QSAR studies. J AOAC Int.

[2] Palczewska A, Neagu D, Ridley M (2013). Using Pareto points for model identification in predictive toxicology. J Cheminform.

[3] Yang SP, Song ST, Tang ZM, Song HF (2003). Optimization of antisense drug design against conservative local motif in simulant secondary structures of HER-2 mRNA and QSAR analysis. Acta Pharmacol Sin.

[4] Liu SS, Liu HL, Yin CS, Wang LS (2003). VSMP: a novel variable selection and modeling method based on the prediction. J Chem Inf Comput Sci.

[5] Soto AJ, Cecchini RL, Vazquez GE, Ponzoni I (2009). Multi-objective feature selection in QSAR using a machine learning approach. QSAR Comb Sci.

[6] Shahlaei M, Madadkar-Sobhani A, Saghaie L, Fassihi A (2012). Application of an expert system based on genetic algorithm- adaptive neuro-fuzzy interference system (GA-ANFIS) in QSAR of cathepsin K inhibitors. Expert Syst Appl.

[7] Teixeira AL, Leal JP, Falcao AO (2013). Random forests for feature selection in QSPR Models—an application for predicting standard enthalpy of formation of hydrocarbons. J Cheminform.

[8] Shahlaei M (2013). Descriptor selection methods in quantitative structure-activity relationship studies: a review study. Chem Rev.

[9] Hewitt M, Ellison CM, Enoch SJ, Madden JC, Cronin MTD (2010). Integrating (Q)SAR models, expert systems and read-across approaches for the prediction of developmental toxicity. Reprod Toxicol.

[10] GramaticaP, Cassani S, Roy PP, Kovarich S, Yap CW, Papa E (2012) QSAR Modeling is not “Push a Button and Find a Correlation”: a case study of toxicity of (benzo‐) triazoleson algae. Mol Inform 31(11–12):817–835 10.1002/minf.201200075 27476736

[11] Palomba D, Martínez MJ, Ponzoni I, Díaz MF, Vazquez GE, Soto AJ (2012). QSPR models for predicting log pliver values for volatile organic compounds combining statistical methods and domain knowledge. Molecules.

[12] Zhang Q, Hughes-Oliver JM, Ng RT (2009). A model-based ensembling approach for developing QSARs. J ChemInf Model.

[13] Cao DS, Xu QS, Liang YZ, Chen X, Li HD (2010). Automatic feature subset selection for decision tree-based ensemble methods in the prediction of bioactivity. Chemometr Intell Lab Syst.

[14] Keim DA, Kohlhammer J, Ellis G, Mansmann F (2010). Mastering the information age-solving problems with visual analytics.

[15] Dietzsch J, Heinrich J, Nieselt K, Bartz D (2009) Spray: a visual analytics approach for gene expression data. In: IEEE (ed) Visual analytics science and technology, 2009. IEEE symposium on VAST 2009, pp 179–186

[16] Santamaría R, Therón R, Quintales L (2008). A visual analytics approachfor understanding biclustering results from microarray data. BMC Bioinform.

[17] Schatz MC, Phillippy AM, Shneiderman B, Salzberg SL (2007). Hawkeye: an interactive visual analytics tool for genome assemblies. Genomebiology.

[18] Gütlein M, Karwath A, Kramer S (2012). CheS–Mapper–Chemical space mapping and visualization in 3D. J Cheminform.

[19] Backman TW, Cao Y, Girke T (2011) ChemMine tools: an online service for analyzing and clustering small molecules. Nucleic Acids Res 39(Web Server issue):W486–W491 10.1093/nar/gkr320 PMC3125754 21576229

[20] Awale M, Van Deursen R, Reymond JL (2013). MQN-mapplet: visualization of chemical space with interactive maps of DrugBank, ChEMBL, PubChem, GDB-11, and GDB-13. J Chem Inf Model.

[21] Gramatica P, Chirico N, Papa E, Cassani S, Kovarich S (2013). QSARINS: A new software for the development, analysis, and validation of QSAR MLR models. J Comput Chem.

[22] Krause J, Perer A, Bertini E (2014). INFUSE: interactive feature selection for predictive modeling of high dimensional data. IEEE Trans Vis Comput Graph.

[23] Ganguly M, Brown N, Schuffenhauer A, Ertl P, Gillet VJ, Greenidge PA (2006). Introducing the consensus modeling concept in genetic algorithms: application to interpretable discriminant analysis. J Chem Inf Model.

[24] Cover TM, Thomas JA (1991). Elements of information theory. Entropy, relative entropy and mutual information.

[25] Kojadinovic I (2005) On the use of mutual information in data analysis: an overview. In: Proceedings to 11th international symposium on applied stochastic models and data analysis, Brest, France, pp 738–747

[26] Soto AJ, Vazquez GE, Stricker M, Ponzoni I (2011). Target-driven subspace mapping methods and their applicability domain estimation. Mol Inform.

[27] Hall M, Frank E, Holmes G, Pfahringer B, Reutemann P, Witten IH (2009). The WEKA data mining software: an update. ACM SIGKDD Explor Newslett.

[28] Katritzky AR, Kuanar M, Fara DC, Karelson M, Acree WE, Solov’ev VP (2005). QSAR modeling of blood:air and tissue:air partition coefficients using theoretical descriptors. Bioorg Med Chem.

[29] Dashtbozorgi Z, Golmohammadi H (2010). Prediction of air to liver partition coefficient for volatile organic compounds using QSAR approaches. Eur J Med Chem.

[30] Abraham MH, Ibrahim A, Acree WE (2007). Air to liver partition coefficients for volatile organic compounds and blood to liver partition coefficients for volatile organic compounds and drugs. Eur J Med Chem.

[31] Audi R (1999). Ockham’s razor, the Cambridge dictionary of philosophy.

[32] Ward M, Sweeney J (2012). Mechanical properties of solid polymers.

[33] Callister WD (2007). Materials science and engineering: an introduction.

[34] Palomba D, Vazquez GE, Díaz MF (2014) Prediction of elongation at break for linear polymers. Chemometrics Intell Lab Syst 139:121–131

[35] Peixun L, Wei L (2009). Current mathematical methods used in QSAR/QSPR studies. Int J Mol Sci.

