# Supplementary material for: Visual analytics in cheminformatics: user-supervised descriptor selection for QSAR methods
Source: J Cheminform. 2015 Aug 19;7:39. doi: 10.1186/s13321-015-0092-4 (PMC4540751; doi:10.1186/s13321-015-0092-4)
Supplement: Additional file 1. — The parameter setup and tables with the predictive accuracy of models used in the case studies. [file 13321_2015_92_MOESM1_ESM.doc]

**Visual Analytics in Cheminformatics: User-Supervised Descriptor Selection for QSAR Methods**

María Jimena Martínez ^1^, Ignacio Ponzoni ^1^, Mónica F. Díaz ^2^, Gustavo E. Vazquez ^3^, Axel J. Soto ^4^

1 Laboratorio de Investigación y Desarrollo en Computación Científica (LIDeCC), Instituto de Ciencias e Ingeniería de la Computación (ICIC), Departamento de Ciencias e Ingeniería de la Computación, Universidad Nacional del Sur, Av. Alem 1253, 8000, Bahía Blanca, Argentina

2 Planta Piloto de Ingeniería Química (PLAPIQUI) - UNS – CONICET Complejo CRIBABB, Co. La Carrindanga km.7, CC 717, Bahía Blanca, Argentina

^3^ Facultad de Ingeniería y Tecnologías, Universidad Católica del Uruguay, Av. 8 de Octubre 2801, CC 11300, Montevideo, Uruguay

^4^ Faculty of Computer Science, Dalhousie University, 6050 University Av., Halifax, Canada

All models were run with the following classifiers and parameters:

**functions: LinearRegression**

attributeSelectionMethod: M5 method

eliminateColinearAttributes: true

ridge: 1.0E-8

**trees: M5P**

buildRegressionTree: false

unpruned: false

useUnsmoothed: false

minNumInstances: 4.0

**functions: MultilayerPerceptron**

nominalToBinaryFilter: true

decay: false

normalizeAttributes: true

normalizeNumericClass: true

reset: true

hiddenLayers: (attributes+classes/2)

learningRate: 0.3

momentum: 0.2

seed: 0

trainingTime: 500

validationThreshold: 20

validationSetSize: 0

In the following three tables, the best predictive accuracy is highlighted in bold.

|  | **Predictive accuracy** | | |
| --- | --- | --- | --- |
| **Model** | **Linear Regression** | **Decision Trees (M5P)** | **Neural Network**  **(Multilayer Perceptron)** |
| **M1 (ALOGP , Mor29u , AMW, Se, Pol)** | R^2^ = 0.79  MAE = 0.16  RMSE = 0.21 | **R^2^ = 0.81**  **MAE = 0.15**  **RMSE = 0.20** | R^2^ = 0.73  MAE = 0.20  RMSE = 0.25 |
| **M2 (ALOGP, SP15, RDF015v, RDF020e, H6v)** | R^2^ = 0.72  MAE = 0.18  RMSE = 0.24 | **R^2^ = 0.76**  **MAE = 0.17**  **RMSE = 0.23** | R^2^ = 0.55  MAE = 0.26  RMSE = 0.34 |
| **M3 (ALOGP, Mor29u, X4Av, ESpm15,Mor31e, Ui)** | **R^2^ = 0.79**  **MAE = 0.16**  **RMSE = 0.21** | **R^2^ = 0.79**  **MAE = 0.16**  **RMSE = 0.21** | R^2^ = 0.70  MAE = 0.23  RMSE = 0.29 |
| **M4 (ALOGP, Mor29u, X4Av, DP06, QZZv, Mor02v, F01[C-C])** | R^2^ = 0.76  MAE = 0.17  RMSE = 0.22 | **R^2^ = 0.79**  **MAE = 0.16**  **RMSE = 0.21** | R^2^ = 0.67  MAE = 0.20  RMSE = 0.32 |

**Table 1:** Predictive accuracy in Weka using 4-fold cross validation.

|  | | | **Predictive accuracy** | |
| --- | --- | --- | --- | --- |
| **Model** | **Linear Regression** | **Decision Trees (M5P)** | | **Neural Network (Multilayer Perceptron)** |
| **M1 (Mn/MW, Sp, RHyDp, ETA_EtaP_F_L)** | R^2^ = 0.20  MAE = 4.63  RMSE = 7.37 | R^2^ = 0.23  MAE = 3.98  RMSE = 6.14 | | **R^2^ = 0.26**  **MAE = 4.62**  **RMSE = 8.14** |
| **M2 (Mn/MW, MDEO-11, D/Dr09, SMTIV)** | R^2^ = 0.20  MAE = 4.39  RMSE = 7.44 | R^2^ = 0.26  MAE = 4.32  RMSE = 7.24 | | **R^2^ = 0.32**  **MAE = 5.94**  **RMSE = 8.31** |
| **M3 (Mn/MW, nHBint4, nHBint10, ETA_dEpsilon_B)** | **R^2^ = 0.56**  **MAE = 4.03**  **RMSE = 6.22** | R^2^ = 0.56  MAE = 4.03  RMSE = 6.22 | | R^2^ = 0.46  MAE = 4.63  RMSE = 7.17 |
| **M4 (Mn/MW, nsCH3, nF6Ring, ALOGP2, RDCHI)** | R^2^ = 0.41  MAE = 4.47  RMSE = 6.77 | **R^2^ = 0.41**  **MAE = 3.94**  **RMSE = 6.75** | | R^2^ = 0.34  MAE = 5.51  RMSE = 8.43 |
| **M5 (Mn/MW, nROH, n6Ring, nHCsatu, ALOGP2)** | R^2^ = 0.37  MAE = 4.18  RMSE = 7.71 | R^2^ = 0.25  MAE = 4.29  RMSE = 7.51 | | **R^2^ = 0.68**  **MAE = 3.28**  **RMSE = 5.78** |
| **M6 (Mn/MW,nP, minHBa, T(O..P), ETA_Epsilon_3)** | R^2^ = -0.04  MAE = 5.97  RMSE = 15.86 | **R^2^ = 0.25**  **MAE = 4.48**  **RMSE = 7.19** | | R^2^ = 0.15  MAE = 5.95  RMSE = 10.74 |
| **M7 (Mn/MW, ETA_dEpsilon_B, C-005, SHaaCH, nHBint9,nCt )** | R^2^ = 0.21  MAE = 4.52  RMSE = 7.50 | **R^2^ = 0.31**  **MAE = 4.19**  **RMSE = 7.20** | | R^2^ = 0.15  MAE = 6.13  RMSE = 8.59 |
| **M8 (Mn/MW, ndssC, minHBint9, MSD, C-004, Mw/Mn (PDI), crosshead speed(CHS))** | R^2^ = 0.18  MAE = 4.41  RMSE = 7.66 | **R^2^ = 0.39**  **MAE = 3.92**  **RMSE = 6.86** | | R^2^ = 0.27  MAE = 8.81  RMSE = 14.79 |
| **M9 (Mn/MW, Pol, Wap, maxHAvin, nHAvin, MWC04)** | **R^2^ = 0.15**  **MAE = 4.92**  **RMSE = 7.88** | R^2^ = 0.09  MAE = 4.87  RMSE = 7.82 | | R^2^ = -0.02  MAE = 5.74  RMSE = 8.69 |
| **M10 (Mn/MW,maxHBint6, ETA_dEpsilon_A, TIC2, ndO, nHdCH2)** | R^2^ = 0.30  MAE = 4.40  RMSE = 7.24 | R^2^ = 0.28  MAE = 4.13  RMSE = 7.22 | | **R^2^ = 0.48**  **MAE = 4.02**  **RMSE = 7.09** |

**Table 2:** Predictive accuracy in Weka using 4-fold cross validation.

|  | **Predictive accuracy** | | |
| --- | --- | --- | --- |
| **Model** | **Linear Regression** | **Decision Trees(M5P)** | **Neural Network (Multilayer Perceptron)** |
| **M3 + CHS (Mn/MW, nHBint4, nHBint10, ETA_dEpsilon_B,CHS)** | R^2^ = 0.57  MAE = 3.98  RMSE = 6.14 | **R^2^ = 0.62**  **MAE = 3.43**  **RMSE = 5.89** | R^2^ = 0.54  MAE = 4.69  RMSE = 6.90 |
| **M3 + CHS - nHBint4 (Mn/MW, nHBint10, ETA_dEpsilon_B,CHS)** | R^2^ = 0.57  MAE = 3.98  RMSE = 6.14 | **R^2^ = 0.69**  **MAE = 3.24**  **RMSE = 5.68** | R^2^ = 0.54  MAE = 4.69  RMSE = 6.90 |

**Table 3:** Predictive accuracy in Weka using 4-fold cross validation.
